# Declines in Pediatric Bacterial Meningitis in the Republic of Benin Following Introduction of Pneumococcal Conjugate Vaccine: Epidemiological and Etiological Findings, 2011–2016

**DOI:** 10.1093/cid/ciz478

**Published:** 2019-09-05

**Authors:** Joseph Agossou, Chinelo Ebruke, Alphonse Noudamadjo, Julien D Adédémy, Eric Y Dènon, Honoré S Bankolé, Mariam A Dogo, Rolande Assogba, Moussa Alassane, Abdoullah Condé, Falilatou Agbeille Mohamed, Gérard Kpanidja, Moutawakilou Gomina, François Hounsou, Basile G Aouanou, Catherine Okoi, Claire Oluwalana, Archibald Worwui, Peter S Ndow, Jean Nounagnon, Jason M Mwenda, Rock A Sossou, Brenda A Kwambana-Adams, Martin Antonio

**Affiliations:** 1 Department of Mother and Child, Faculty of Medicine, University of Parakou, Parakou, Benin; 2 Borgou Regional University Teaching Hospital, Parakou, Benin; 3 World Health Organization (WHO) Collaborating Centre for New Vaccines Surveillance, Medical Research Council Unit The Gambia at London School of Hygiene and Tropical Medicine, Fajara, Banjul; 4 Service National de Laboratoire Sante Publique, Cotonou; 5 Saint Jean de Dieu Hospital of Tanguieta, Benin; 6 WHO Regional Office for Africa, Brazzaville, Republic of Congo; 7 WHO Country Office, Cotonou, Benin; 8 Microbiology and Infection Unit, Warwick Medical School, University of Warwick, Coventry, United Kingdom

**Keywords:** Benin, pediatric, bacterial meningitis, pneumococcus, vaccines

## Abstract

**Background:**

Pediatric bacterial meningitis (PBM) remains an important cause of disease in children in Africa. We describe findings from sentinel site bacterial meningitis surveillance in children <5 years of age in the Republic of Benin, 2011–2016.

**Methods:**

Cerebrospinal fluid (CSF) was collected from children admitted to Parakou, Natitingou, and Tanguieta sentinel hospitals with suspected meningitis. Identification of *Streptococcus pneumoniae* (pneumococcus), *Haemophilus influenzae*, and *Neisseria meningitidis* (meningococcus) was performed by rapid diagnostic tests, microbiological culture, and/or polymerase chain reaction; where possible, serotyping/grouping was performed.

**Results:**

A total of 10 919 suspected cases of meningitis were admitted to the sentinel hospitals. Most patients were 0–11 months old (4863 [44.5%]) and there were 542 (5.0%) in-hospital deaths. Overall, 4168 CSF samples were screened for pathogens and a total of 194 (4.7%) PBM cases were confirmed, predominantly caused by pneumococcus (98 [50.5%]). Following pneumococcal conjugate vaccine (PCV) introduction in 2011, annual suspected meningitis cases and deaths (case fatality rate) progressively declined from 2534 to 1359 and from 164 (6.5%) to 14 (1.0%) in 2012 and 2016, respectively (*P* < .001). Additionally, there was a gradual decline in the proportion of meningitis cases caused by pneumococcus, from 77.3% (17/22) in 2011 to 32.4% (11/34) in 2016 (odds ratio, 7.11 [95% confidence interval, 2.08–24.30]). *Haemophilus influenzae* meningitis fluctuated over the surveillance period and was the predominant pathogen (16/34 [47.1%]) by 2016.

**Conclusions:**

The observed decrease in pneumococcal meningitis after PCV introduction may be indicative of changing patterns of PBM etiology in Benin. Maintaining vigilant and effective surveillance is critical for understanding these changes and their wider public health implications.

## Teaser

We conducted sentinel pediatric bacterial meningitis surveillance following pneumococcal conjugate vaccine introduction in Benin during 2011–2016. A total of 194 cases were confirmed, predominantly caused by pneumococcus (50.5%). Overall, meningitis and pneumococcal meningitis cases progressively declined during surveillance.

Invasive bacterial disease, particularly bacterial meningitis, remains an important cause of morbidity and mortality in developing countries [1]. Globally, the burden of bacterial meningitis is heaviest across the African meningitis belt, a region in sub-Saharan Africa spanning from Senegal to Ethiopia. Outbreaks of epidemic bacterial meningitis occur in this region frequently and are associated with high death tolls [2–4]. For instance, an outbreak affecting 10 countries, including the Republic of Benin, occurred in 2012, with an estimated case fatality rate (CFR) of 8.2% [5]. The public health significance of bacterial meningitis extends beyond mortality, as survivors are often left with life-changing sequelae [6, 7]. Previous reports estimated that half of all surviving children have lifelong neurologic impairment including deafness, learning disabilities, and seizures [8, 9].

Bacterial meningitis is predominately caused by 3 pathogens, *Neisseria meningitidis* (meningococcus), *Streptococcus pneumoniae* (pneumococcus), or *Haemophilus influenzae*, and can be successfully prevented by immunization. Thus, organizations such as Gavi, the Vaccine Alliance have funded the introduction of the pneumococcal conjugate vaccines (PCVs), *H. influenzae* type b (Hib) vaccine, and a meningococcal conjugate vaccine (MenAfriVac) in a number of countries across sub-Saharan Africa [10–12]. Consequently, it is expected that the magnitude and severity of meningitis outbreaks in this region should begin to decline. However, monitoring the impact of vaccine introduction remains a challenge in these countries, mostly due to weak disease surveillance systems [13].

Therefore, as part of the World Health Organization (WHO) Invasive Bacterial Vaccine-Preventable Disease (IB-VPD) surveillance network, the Medical Research Council Unit The Gambia (MRCG) was designated as a regional reference laboratory and a WHO Collaborating Centre (CC) to support the surveillance of pediatric bacterial meningitis (PBM) across countries in West and Central Africa. This includes the Republic of Benin, where the Hib vaccine and 13-valent PCV (PCV13) were introduced in 2005 and 2011 with coverage as high as 90% and 75%, respectively [14]. In addition, a mass MenAfriVac vaccination campaign took place in the northern parts of the Republic of Benin in late 2012 [15, 16]. Here, we describe the epidemiology and etiology of bacterial meningitis in children <5 years old, after longitudinal surveillance from 2011 through 2016, in the Republic of Benin.

## METHODS

### Surveillance Design

The Republic of Benin is located in Western Africa, with a total land area of 112 622 km^2^ and an estimated population of 11.5 million. Temperatures range from 24°C to 31°C with a dry season occurring from December to April and a rainy season from April to late July, then September to November, thus a bimodal rainfall pattern. National prevalence for human immunodeficiency virus (HIV) in Benin during the surveillance period was between 1.0% (2016) and 1.2% (2011) [17].

Hospital-based surveillance was conducted at 3 hospitals, the Centre Hospitalier Universitaire Départemental du Borgou/Alibori in Parakou, L’hopital Saint Jean de Dieu de Tanguieta and the Centre Hospitalier Départemental de l’Atacoralocated in Natitingou, located in northern Benin. These hospitals serve as referral centers, receiving patients referred from other health facilities within and outside the city as well as self-referred patients who are seen at the emergency department or the outpatient clinics. Both hospitals also provide 24-hour emergency medical care to the public including for patients with suspected meningitis.

### Case Enrollment

Standardized guidelines including case definitions and laboratory protocols developed by the WHO were applied during the surveillance period [18]. All cases of suspected meningitis in children aged <5 years admitted to the sentinel hospitals during the surveillance period were enrolled. Cerebrospinal fluid (CSF) was collected at the time of admission or soon after, as part of routine clinical evaluation. The WHO clinical case definition for suspected meningitis, defined as sudden onset of fever (>38°C axillary or >38.5°C rectal temperature), and 1 or more of any the following clinical symptoms: altered consciousness, neck stiffness, sensitivity to light, and bulging of the fontanelle if <1 year old, was applied throughout the surveillance period.

### Laboratory Diagnostics at the Sentinel Hospitals

CSF samples were sent to the Service National de Laboratoire Sante Publique in Cotonou, southern Benin, after macroscopic and microscopic examination of CSF was performed in the sentinel hospital laboratory. White blood cell (WBC) counts were performed using a Neubauer counting chamber, glucose concentrations were determined using the glucose oxidase method, and CSF protein levels were determined using the trichloroacetic acid turbidimetric method, as previously described [19, 20]. Identification of pneumococcus, *H. influenzae*, and meningococcus was determined by Gram staining, colony morphology on appropriate agar plates, and rapid diagnostic tests (RDTs). The RDTs used included the Pastorex meningitis kit (Bio-Rad, United Kingdom) for pneumococcus, Hib, and meningococcal serogroup A, B, C, Y, and W antigen detection and the BINAX NOW kit (Alere Inc) for pneumococcal antigen detection. All procedures were performed according to WHO standards [21].

### Laboratory Diagnostics at the WHO CC, MRCG

Viable bacterial isolates and a proportion of CSF culture-negative samples were sent to the WHO CC at MRCG for further testing. Identification of pneumococcus, *H. influenzae*, and meningococcus bacterial isolates were confirmed by colony morphology. Pneumococcal isolates were confirmed with optochin tests (5-μg optochin disk; Oxoid, Basingstoke, United Kingdom), and the analytical profile index (API NH; bioMérieux, United Kingdom) was used for confirmation of meningococcus and *H. influenzae* isolates. Pneumococcal isolates were serotyped by latex agglutination using latex antisera (Statens Serum Institut, Copenhagen, Denmark) for detecting ≥90 pneumococcal serotypes, whereas *H. influenzae* isolates were serotyped using antisera for serotypes a to f (Murex Biotech, Dartford, United Kingdom), as described previously [22].

Real-time polymerase chain reaction (qPCR) was used for identification of pneumococcus, *H. influenzae*, and meningococcus, as previously described [2]. In brief, species-specific qPCR was performed targeting the autolysin gene (*lytA*), protein D encoding gene (*hpd*), and Cu, Zn superoxide dismutase gene (*sodC*) for the detection of pneumococcus, *H. influenzae*, and meningococcus, respectively. An RNase P gene assay was performed concurrently to assess the integrity of the CSF samples. Cycle threshold (Ct) values of ≤36 were considered positive results.

*Haemophilus influenzae* serotyping was performed by qPCR targeting *acsB*, *bcsB*, *ccsD*, *dscE*, *ecsH*, and *bexD* genes for the identification of serotypes a–f, respectively. Meningococcal serogroups were determined targeting *sacB*, *synD*, *synE*, *synG*, *xcbB*, and *synF* for identification of serogroups A, B, C, W, X, and Y, respectively. Ct values of ≤36 were considered positive. For pneumococcal serotyping, nucleic acid extraction of CSF samples was performed using the Qiagen DNA kit (Qiagen, Crawley, United Kingdom), and a sequential triplex qPCR assay, which detects 21 serotypes commonly isolated in Africa, was performed. Ct values of ≤36 were considered positive. Pneumococci that could not be typed, with Ct values >32, were further subjected to sequential conventional multiplex serotyping PCRs.

### Statistical Analysis

All statistical analysis was carried out using Stata software version 12 (StataCorp, College Station, Texas). Associations between selected clinical symptoms and sequelae or pathogen detection by qPCR were determined using a χ ^2^ test. A *P* value <.05 indicates statistical significance. Significant associations were subjected to logistic regression to estimate the direction of association.

**Ethical Considerations**

Ethical approval was not a requirement in Benin for routine meningitis surveillance, including drug susceptibility testing of collected isolates, as this was approved as part of the routine diagnostic algorithm at the Ministry of Health. However, informed consent was sought from the parents or guardians of the surveillance participants. Additionally, the surveillance received overarching ethical approval (SCC1188) by the joint Medical Research Council (MRC)/The Gambia government ethics board that allowed the analysis of collected West African isolates at MRCG.

## RESULTS

### Demographic and Clinical Characteristics

A total of 10 919 children with suspected meningitis were admitted at the sentinel hospitals from 2011 through 2016. Overall, 5888 (53.9%) patients were male and the median age of patients was 22 months (interquartile range, 1–23 months; Table 1). More than two-fifths of patients with suspected meningitis (4863 [44.5%]) were aged 0–11 months.

**Table 1.** Summary of Patient Characteristics With Suspected Bacterial Meningitis in the Republic of Benin, 2011–2016

Characteristic	All Suspected Cases, No. (%)	CSF Specimen Collected, No. (%)	CSF Specimen Not Collected, No. (%)	*P* Value^a^
Age				
0–11 mo	4863 (44.5)	1788 (42.9)	3075 (45.5)	.26
12–23 mo	2038 (18.7)	772 (18.5)	1266 (18.8)	
24–59 mo	3826 (35.0)	1496 (35.9)	2330 (34.5)	
Unknown	192 (1.8)	112 (2.7)	80 (1.2)	
Sex				
Male	5888 (53.9)	2224 (53.4)	3664 (54.3)	.38
Female	4929 (45.1)	1888 (45.3)	3041 (45.0)	
Unknown	102 (0.9)	56 (1.3)	46 (0.7)	
Antibiotics before admission				
Yes	997 (9.1)	472 (11.3)	525 (7.8)	.08
No	4641 (42.5)	1837 (44.1)	2804 (41.5)	
Unknown	5281 (48.4)	1859 (44.6)	3422 (50.7)	
Outcome				
Discharged alive	7652 (70.1)	2762 (66.3)	4890 (72.4)	.07
Died	542 (5.0)	131 (3.1)	411 (6.1)	
Unknown	2725 (25.0)	1275 (30.6)	1450 (21.5)	
Total suspected cases^b^	10 919 (100.0)	4168 (100.0)	6751 (100.0)	

Abbreviation: CSF, cerebrospinal fluid.

^a^Comparison between suspected cases from whom CSF specimens were collected and those in whom no specimen was collected.

^b^Suspected cases include cases that were defined as probable as per World Health Organization case definition guidelines [18].

In-hospital deaths accounted for 542 patients (CFR, 5.0%), whereas 7652 (70.1%) children were discharged from hospital alive and discharge status data were unavailable for 2725 (24.9%). Of these 542 patients, children aged 24–59 months accounted for most deaths (241 [44.5%]), followed by those aged 0–11 months (184 [33.9%]). There were more deaths in male children than in females (285 [52.6%] vs 252 [46.5%]; *P* = .04). Additionally, only 58 of 7652 (0.8%) patients who survived were reported to have lasting sequelae at the time of hospital discharge, including hearing loss and limb weakness, whereas 2128 (27.8%) had no sequelae and 5466 (71.4%) had missing data on sequelae.

**Figure 1.**
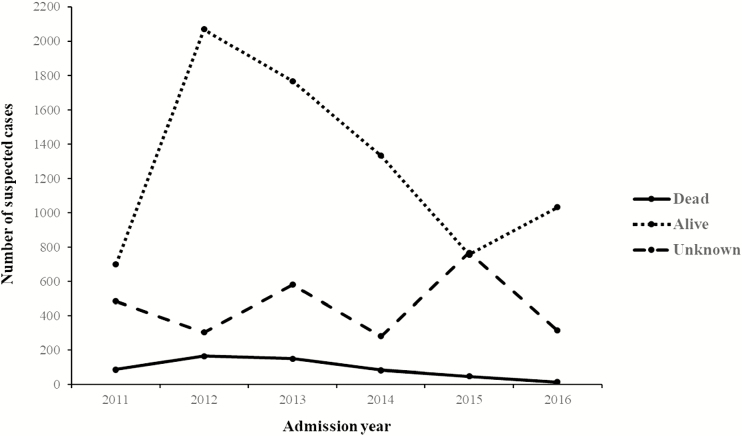
Yearly distribution of outcomes for suspected meningitis cases admitted to hospital in the Republic of Benin, 2011–2016.

**Figure 2.**
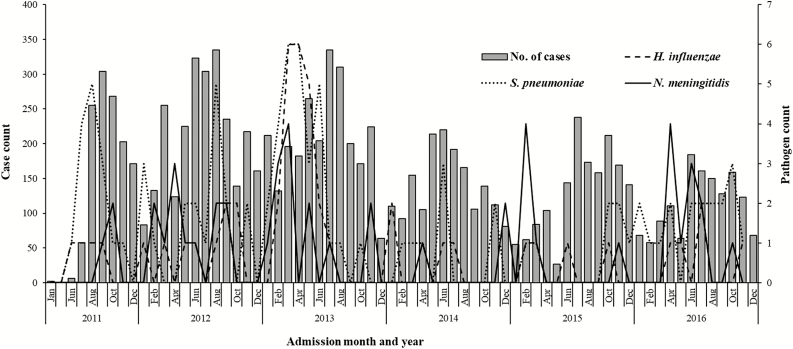
Distribution of pathogens isolated and suspected meningitis cases admitted to hospital in the Republic of Benin by month and year for the period 2011–2016.

The annual distribution of admission outcomes over the surveillance period showed a progressive decline in both the total number of suspected cases per year (2534 in 2012 to 1359 in 2016) and the number of deaths (164 [CFR, 6.5%] in 2012 to 14 [CFR, 1.0%] in 2016; *P* < .001), following the introduction of PCV13 in 2011 (Figure 1). Analysis of cases admitted by month highlighted an annual pattern of admissions, peaking during the cooler dry season from July to September (Figure 2).

A total of 4168 CSF samples from patients with suspected PBM were tested and a bacterial pathogen was detected in 194 (4.7%), confirmed to be either pneumococcus, *H. influenzae*, or meningococcus. The majority of these CSF samples (74.6% [3110/ 4168]) were reported to be clear, whereas 4.4% (182) were turbid (Table 2). However, the proportion of turbid CSF specimens that had a pathogen detected was up to 10 times higher than in CSF samples with other appearances including, clear, blood-stained, and xanthochromic (48.9% in turbid CSF vs <5% for other CSF specimen types). Additionally, the proportion of pathogens identified was highest in CSF samples with WBC counts of >100 cells/μL (14.0%) and lowest in CSF samples with lower WBC counts of <10 cells/μL (2.7%).

**Table 2.** Distribution of Cerebrospinal Fluid Characteristics and Proportion of Pathogen Identified by Any Laboratory Method in the Republic of Benin, 2011–2016

Characteristic	No. (%)	No. of Pathogens (%)
CSF appearance		
Clear	3110 (74.6)	78 (2.5)
Turbid/cloudy	182 (4.4)	89 (48.9)
Xanthrochromic	237 (5.7)	8 (3.4)
Blood stained	413 (9.9)	8 (1.9)
Unknown	226 (5.4)	11 (4.9)
WBC count, cells/μL		
≤10	3006 (72.1)	79 (2.6)
>10 to 100	217 (5.2)	19 (8.8)
>100	543 (13.0)	76 (14.0)
Unknown	402 (9.6)	20 (5.0)
Protein, mg/dL		
≤100	439 (10.5)	26 (5.9)
>100	238 (5.7)	11 (4.6)
Unknown	3491 (83.8)	157 (4.5)
Glucose, g/dL		
<40	545 (13.1)	31 (5.7)
≥40	1792 (43.0)	28 (1.6)
Unknown	1831 (43.9)	135 (7.4)
Total CSF-tested suspected cases	4168	194 (4.7)

Abbreviations: CSF, cerebrospinal fluid; WBC, white blood cell.

### Pathogen Distribution

Of the 194 pathogens detected, pneumococcus was the predominant pathogen, accounting for 98 cases (50.5%). *Haemophilus influenzae* and meningococcus accounted for a quarter each (48 cases [24.7%]) of all positive samples (Table 3). When disaggregated by year, a gradual decline in the proportion of pathogens identified was observed. Pneumococcus decreased from 77.3% (17/22) in 2011 to 32.4% (11/34) in 2016 (odds ratio, 7.11 [95% confidence interval, 2.08–24.30]). In contrast, the proportion of *H. influenzae* appeared to fluctuate over the same time period and, by 2016, it was the predominant pathogen (16/34 [47.1%]) identified among positive CSF samples (Table 3).

**Table 3.** Yearly Distribution of Bacterial Meningitis Pathogens in the Republic of Benin, 2011–2016

Year	CSF Samples Tested, No.	Pathogens Identified	Pneumococcus	*Haemophilus influenzae*	Meningococcus
		No.	No. (%)^a^	No. (%)^a^	No. (%)^a^
2011	184	22	17 (77.3)	3 (13.6)	2 (9.1)
2012	105	39	21 (53.8)	5 (12.8)	13 (33.3)
2013	283	64	29 (45.3)	16 (25.0)	19 (29.7)
2014	1069	18	10 (55.6)	4 (22.2)	4 (22.2)
2015	1252	17	10 (58.8)	4 (23.5)	3 (17.6)
2016	1275	34	11 (32.4)	16 (47.1)	7 (20.6)
Total	4168	194	98 (50.5)	48 (24.7)	48 (24.7)

Abbreviation: CSF, cerebrospinal fluid.

^a^Percentages for each pathogen were calculated based on the total number of pathogens identified for each year.

Pneumococcal serotyping was performed on 40 of 98 positive CSF samples collected from 2011 to 2016. Prevalence of vaccine-type serotypes (VTs) and nonvaccine-type serotypes (NVTs) fluctuated from year to year, thus showing no clear pattern of NVT replacement post–PCV13 introduction. Of the 40 pneumococcal isolates for which serotyping was performed, proportions of VT and NVT serotypes identified during the surveillance period were, respectively, 66.7% and 33.3% in 2011; 54.5% and 45.5% in 2012; 66.7% and 33.3% in 2013; 40.0% and 60.0% in 2014; 83.0% and 17.0% in 2015; and 33.3% and 66.7% in 2016. The predominant VTs were serotypes 6 (10/59 [16.9%]) and serotypes 14 and 18C (3/40 each [7.5%]), whereas the predominant NVT was serotype 12 (5/40 [12.5%]). Of the 98 cases of pneumococcal meningitis, only 4 (4.1%) had received PCV13 vaccination, whereas vaccination data for the remaining 94 (95.9%) were unavailable. Of these 4, serotyping was confirmed for 1 case and was found to be an NVT (35B).

Serotyping was performed on 15 of 48 of *H. influenzae*–positive CSF samples collected from 2013 to 2016. A total of 3 Hib isolates were identified, 1 in 2013 and 2 in 2016. Hib vaccine data for these cases were unavailable. Meningococcal serogrouping was performed on 16 of 48 positive CSF samples collected from 2012 to 2016. Approximately, one-third of meningococcal isolates were nongroupable by PCR (5/16 [31.3%]), whereas the majority of those grouped belonged to the W (4/16 [25.0%]), X (3/16 [18.8%]), and A (2/16 [12.5%]) serogroups.

## DISCUSSION

Here, we highlight bacterial causes of pediatric meningitis over a 6-year period, including the period following the introduction of Hib and PCVs in the Republic of Benin. In particular, we report on progressive declines in the total numbers of meningitis cases and in the prevalence of pneumococcal meningitis following introduction of PCVs in the Republic of Benin. Also, relevant epidemiological findings from PBM cases were reviewed, thus, providing a clinical and demographic context for interpretation of the laboratory results.

Pneumococcus was the predominant pathogen identified throughout the surveillance period, even though the prevalence was seen to progressively decline in the post-PCV era. This decrease in prevalence of pneumococcal meningitis appeared to mirror the progressive declines in total numbers of meningitis cases reported during the same time period. Declining rates of invasive pneumococcal disease, including pneumococcal meningitis, following PCV introduction have been reported elsewhere [23–25]. In these earlier studies, the decline in disease was attributed to decreases in prevalence of pneumococcal VT serotypes, following PCV introduction [23, 25]. The same trend was not observed in the current report, where the number of pneumococcal meningitis cases caused by both VT and NVT serotypes fluctuated by year. Also, we were not able to assess the likely impact of other possible explanations for the declines in overall meningitis cases observed during this surveillance period, such as any year-to-year changes in the patterns of viral infections or possible impact of introduction of antiretroviral therapy programs across the country. The prevalence of HIV in Benin remained relatively low (1.2% in 2011; 1.0% in 2016) throughout the surveillance period, and it is unlikely that this may have had an influence on the observed declines in meningitis cases during this period [17]. Although it has not been possible to rule out the influence of other interventions on prevalence of meningitis during this surveillance period, our findings provide some indication of the likely impact of PCV introduction on meningitis in this setting. This report also highlights the need for ongoing surveillance to provide robust data for the evaluation of vaccine impact in the post-PCV era.

Serotype replacement of VT serotypes with NVT serotypes has been reported in other settings [26, 27]. Our results did not provide clear evidence of serotype replacement following PCV introduction. However, less than half (41.7%) of the recovered pneumococcal isolates were serotyped. Thus, the extent to which our findings represent the true proportions of pneumococcal meningitis caused by VT and NVT serotypes in a pre- and post-PCV era is limited. Serogroup 12 was identified as the predominant NVT during the surveillance period. Serotype 12F has been reported to be an important replacement serotype and has been associated with epidemic outbreaks in parts of North America and Europe [28, 29]. More importantly, this serotype has similar characteristics with serotype 1 and is commonly identified in invasive disease [28]. Given that serotype replacement has been shown in other countries within West Africa, where PCV has been introduced, our results indicate the need for continued monitoring of any changes in patterns of the predominant circulating pneumococcal serotypes and their potential to cause epidemics [23].

In contrast to declines in pneumococcal meningitis, prevalence of *H. influenzae* meningitis fluctuated during this surveillance period, a finding that was unexpected, given the earlier introduction of the Hib conjugate vaccine in Benin in 2005 [14]. Reports from elsewhere have shown significant reductions in Hib-associated diseases following vaccination [30]. We note that only 3 cases of Hib meningitis were identified between 2013 and 2016. These small numbers of Hib meningitis cases therefore make any suggestion of an increase in *H. influenzae* meningitis unlikely in this context. One report from The Gambia showed that there was reemergence of 5 cases of Hib disease in 2005–2006, 7 years after introduction of Hib vaccine in the country [31]. In that report, no evidence was found of the emergence of a hypervirulent strain, and the cause of the reemergence of Hib disease was not clear. In the current report, vaccination status of the 3 cases of Hib meningitis identified could not be established. It remains unclear if these 3 meningitis cases represent unvaccinated cases, a failure of the vaccine, or emergence of vaccine escape variants of the pathogen [32]. Nonetheless, these findings reinforce the need for continued disease surveillance following vaccine introduction both to assess vaccine impact and to enable early detection of any changing patterns in disease epidemiology.

Our results highlighted a seasonal pattern of the incidence of suspected bacterial meningitis cases within the Republic of Benin. The annual peaks during July to September contrast with previous reports on seasonality of meningitis in sub-Saharan Africa, where peaks in the dry months of November to March were observed [33]. This difference may have been influenced by a second dry season observed in the Republic of Benin from late July to early September. In addition, most other reports of disease seasonality are related to epidemic meningitis, which is different to the data presented in this paper. It is probable that our findings may be indicative of a different disease epidemiology in Benin and may be helpful in guiding the timing of interventions for effective control of meningitis.

Globally, rates of mortality from bacterial meningitis have varied depending on the regions of the world. Mortality in industrialized country setting has been much lower in comparison to reports from developing country settings such as in Africa and Latin America, where mortality rates as high as 37% have been recorded and the meningitis disease burden is disproportionately heavier [34–36]. Also, when pathogen-specific mortality is considered, pneumococcal meningitis has been reported to have the highest CFR globally, with rates of up to 45% in Africa [37–40]. Thus, our estimated CFR of 5% appears low in comparison to these previous reports from similar geographical settings. However, our estimate has been generated from available routine medical records, which were often incomplete. For example, the outcome at discharge status was unavailable for 25% of patients with suspected meningitis. Thus, the estimate presented in this report could potentially represent an underestimate of the true CFRs observed during the surveillance period.

Young children have previously been identified as having a higher risk of meningitis and of poorer outcomes in comparison to other age groups [41]. Similar findings were observed during our surveillance, highlighting the need for prevention and treatment strategies that are targeted at these high-risk groups. These strategies include the use of vaccines that are immunogenic in infancy and the application of case detection protocols that enable early identification of bacterial meningitis among young children. In addition, the risk of bacterial meningitis has been shown to be higher in people living with HIV, with 1 report showing an increased risk of as much as 8.3 times than seen in the general population [42, 43]. Also, etiological agents of meningitis in persons infected with HIV have been found to differ from those in HIV-uninfected individuals [42, 44]. As a low-HIV setting with national HIV prevalence rates of between 1.0% and 1.2% reported during the surveillance period in Benin, the impact of HIV on meningitis would be expected to be much lower than would be seen in a setting of high HIV prevalence [17]. However, the current report is unable to directly assess the influence of HIV on meningitis risk or on the etiological agents identified, as data on HIV status of children included in this surveillance were unavailable.

We acknowledge some limitations to our surveillance, within the Republic of Benin, that may limit generalization of the findings. For example, surveillance was only carried out within the context of routine clinical settings, and both clinical and demographic data were incomplete for some key outcomes of interest. In particular, data on discharge outcomes, including sequelae and PCV and Hib vaccination data, were unavailable for a large percentage of the children included in this survey, thus limiting our ability to assess the relevance of these factors to the risk for meningitis. Also, the numbers of reported cases in the early years of the surveillance were quite small, CSF samples were not available for analysis for more than half of the patients enrolled at sentinel hospitals, and serotyping/grouping analysis was limited. However, our results indicate that the children from whom CSF samples were collected and who have been included in this analysis were demographically similar to those suspected cases for whom a CSF specimen was not available. Nonetheless, continued disease surveillance using strictly monitored standards, as applied by the WHO CC, would allow robust data to be collected in the long term and aid our understanding of the observed changes.

## CONCLUSIONS

Overall, pneumococcus has remained an important cause of childhood meningitis in the Republic of Benin, even though there was a progressive decline in its prevalence and relative contribution among other meningitis-causing pathogens in the post-PCV era. Over the same period, a concomitant increase in the prevalence of *H. influenzae* disease, with only 3 cases of Hib meningitis identified, suggests the need for closer monitoring to determine any changes in disease patterns a decade after introduction of the Hib vaccine. This report highlights a clear need for ongoing surveillance for early detection of changing patterns to the etiology and epidemiology of PBM in the West Africa subregion.

## Notes

***Invasive Bacterial Disease (IBD) Writing Group members.*** Brenda Kwambana-Adams, Madikay Senghore, Effua Usuf, Archibald Worwui, Egere Uzochukwu, Akram Zaman, Catherine Okoi, Florian Gehre, Leopold Tientcheu, Nuredin Ibrahim Mohammed, Felix Dube, Peter Ndow, Sambou Suso, Sheikh Jarju, Dam Khan, Chinelo Ebruke, Rowan Bancroft, Jason M. Mwenda, and Martin Antonio.

***Author contributions.*** M. A. and J. M. M. established the World Health Organization (WHO)/Regional Office for Africa (AFRO) support for the Paediatric Bacterial Meningitis Surveillance Network in West Africa. M. A. supervised the overall network including setting up the sentinel surveillance system. J. A., A. N., F. H., A. G. B., and J. N. clinically investigated and recruited the patients at the sentinel sites, collected demographic data and performed microbiological testing at sentinel sites, and shipped cerebrospinal fluid and bacterial isolates to WHO Collaborating Centre (CC) Medical Research Council Unit The Gambia (MRCG). Confirmatory testing and molecular analysis at MRCG was performed by C. Ok., C. Ol., A. W., and P. S. N. and was supervised by B. K. A. and M. A.; C. E., B. K. A., and M. A. developed the analysis plan and contributed to the analysis and interpretation of data along with the IBD writing group. C. E., B. K. A., and M. A. drafted the manuscript. All authors contributed to the interpretation of the findings and the writing of the final manuscript.

***Acknowledgments.*** The authors thank the World Customs Organization of Benin and the WHO Intercountry Support Teams for coordination, advice, and support throughout the surveillance period; the surveillance participants and their families in Benin; the staff members and students at the WHO CC MRCG; and the IBD Writing Group for their advice and input. The data sets supporting the conclusions of this article are included within the article.

***Financial support.*** Financial support for sentinel site surveillance was provided by the Benin Ministry of Health, Gavi - the Vaccine Alliance, through a grant to the WHO for the African Paediatric Bacterial Meningitis Surveillance Network.

***Supplement sponsorship.*** This supplement was supported with funds from Gavi, the Vaccine Alliance through The World Health Organization and the CDC Foundation, and The Medical Research Council Unit The Gambia at the London School of Hygiene and Tropical Medicine.

***Potential conflicts of interest.*** The authors: No reported conflicts of interest. All authors have submitted the ICMJE Form for Disclosure of Potential Conflicts of Interest. Conflicts that the editors consider relevant to the content of the manuscript have been disclosed.
